# Faster-and-tighter nitrogen cycle supports mature forest productivity under elevated CO_2_

**DOI:** 10.1126/sciadv.aed2732

**Published:** 2026-07-29

**Authors:** Manon Rumeau, Carolina Mayoral, Fotis Sgouridis, Michaela K. Reay, Yolima Carrillo, Richard J. Norby, Liz Hamilton, Iain P. Hartley, Gianni Micucci, Emma Sayer, A. Rob Mackenzie, Sami Ullah

**Affiliations:** ^1^ Birmingham Institute of Forest Research, University of Birmingham, Birmingham B15 2TT, UK.; ^2^ School of Geography, Earth and Environmental Science, University of Birmingham, Birmingham B15 2TT, UK.; ^3^ School of Geographical Sciences, University of Bristol, Bristol, UK.; ^4^ Organic Geochemistry Unit, School of Chemistry, University of Bristol, Bristol, UK.; ^5^ Hawkesbury Institute for the Environment (HIE), Western Sydney University, Richmond, Australia.; ^6^ Department of Ecology and Evolutionary Biology, University of Tennessee, Knoxville, TN, USA.; ^7^ Geography, Faculty of Science, Environment and Economy, University of Exeter, Exeter, UK.; ^8^ Department of Civil Engineering, McGill University, Montreal H3A 0C3, Canada.; ^9^ Institute of Botany, Ulm University, Ulm, Germany.; ^10^ Lancaster Environment Centre, Lancaster, UK.

## Abstract

Rising atmospheric carbon dioxide (CO_2_) concentration could enhance forest carbon (C) uptake, but nitrogen (N) is thought to progressively constrain this effect. Yet, significant biomass gains were demonstrated in a mature oak forest after 6 years of Free Air CO_2_ Enrichment. This study investigates whether changes in soil N fluxes explain these gains. We show that enhanced N mineralization and ecosystem N conservation support increased productivity under elevated CO_2_ (eCO_2_). In situ net and gross N ammonification was enhanced by ∼30%, with a pronounced effect at oak budburst. Higher N ammonification rates were associated with increases in both fine root biomass and soil respiration. Gross nitrification was reduced, revealing a faster-yet-tighter cycle facilitated by plant-soil interactions. Hence, forest N cycling can adapt to support C uptake under eCO_2_, but in the longer term, this capacity may be constrained by soil-accessible organic N stocks and reductions in anthropogenic N deposition.

## Teaser

Elevated CO_2_ drives a faster-and-tighter N cycle—trees boost belowground C allocation to mine for soil N and sustain growth.

## INTRODUCTION

The anticipated increase in terrestrial plant growth through the carbon dioxide (CO_2_) fertilization effect under rising atmospheric CO_2_ concentration (*1*) has been challenged by some Free Air CO_2_ Enrichment (FACE) experiments in which trees experienced no growth enhancement (*2*, *3*) or increases that were not sustained (*4*). Increased growth under elevated CO_2_ (eCO_2_) can be constrained by progressive nitrogen (N) limitation—a natural phenomenon in which N becomes increasingly sequestered in plant biomass and soil organic matter (SOM) (*5*). Nevertheless, new evidence from a FACE experiment (BIFoR-FACE) in a mature English Oak (*Quercus robur* L.)–dominated forest revealed significant carbon (C) biomass gains after 6 years of eCO_2_ exposure (*6*). This increase in tree woody biomass was associated with an up-regulation of photosynthetic capacity (*7*), an increase in root C exudation (*6*), and an increase in leaf mass per unit area without affecting the leaf C:N ratio (*8*). These findings suggest that despite the mature and late successional state of this forest, progressive N limitation has not yet occurred. Understanding how trees meet their N requirements to enhance biomass C gains under eCO_2_ is critical to predict the long-term response of temperate forests to eCO_2_ and reduce uncertainties in Earth System Models (*9*), especially given that previous experiments have reported contrasting results (*2*, *3*, *10*, *11*).

Investigating N availability in soils under eCO_2_ is challenging as it requires integrating internal N cycling with external N fluxes and with ecosystem N demands. Yet, research to date has primarily focused on the stimulation of N gross ammonification from SOM through increased C allocation belowground under eCO_2_. Enhanced gross ammonification was observed in a young tree plantation at Aspen-FACE (*12*) and during the first 4 years at BIFoR-FACE (*13*). However, no effects were detected in the young pine and sweetgum forests at Duke FACE and Oak Ridge FACE (*11*), respectively, nor in mature eucalyptus forest at EucFACE (*14*) and the mature spruce forest at the Swiss Canopy FACE (*15*). These contrasting responses suggest that the effect of eCO_2_ on gross ammonification depend upon the initial nutrient limitation or upon tree N acquisition strategies (*16*, *17*). Furthermore, enhanced SOM decomposition may only delay N limitation as easily accessible SOM-N or SOM-N content in general may become depleted with time, given that more N will be sequestered in long-term biomass pools (i.e., woody tissues) (*18*, *19*). On the contrary, higher new N inputs or a reduction of ecosystem N losses has the potential to alleviate N limitation in the long term by increasing total ecosystem N stocks (*5*). Asymbiotic biological N fixation (BNF) usually provides low input of new N in temperate forests but could theoretically be up-regulated under eCO_2_ because of higher C availability in soil (*20*), especially in organic horizons that function largely as rhizosphere environments resulting from high fine-root density. However, previous work in forest ecosystems has found no overall effect of eCO_2_ on BNF (*21*). New N is also provided via atmospheric reactive N (Nr) deposition from pollution, which can cause forest decline but also alleviate N limitation of tree growth in mature forests (*22*). Nr deposition is declining and is projected to further decline in many regions because of effective environmental policies limiting NO*_x_* emissions (*23*, *24*). Future Nr decline will likely alter the N status of forests and therefore need to be considered in future projections of N availability. Last, N conservation mechanisms to support biomass growth under eCO_2_ could reduce ecosystem N losses via gaseous emissions and leaching, but these are poorly characterized, especially in temperate forests. For example, research on 30-year-old trees at Duke FACE found no significant changes in nitrous oxide (N_2_O) emissions despite seasonal variations (*25*). By contrast, potential N_2_O emissions from root-free soils incubated under laboratory conditions were enhanced under eCO_2_ at BIFoR-FACE (*13*). To our knowledge, the effects of eCO_2_ on N_2_ emissions have not yet been studied, hindering a comprehensive assessment of soil N cycling (*26*). Hence, there is an urgent need for an integrated assessment of N fluxes under eCO_2_.

Herein, we investigated key internal and external N cycling processes at the BIFoR-FACE facility in Central England across different periods of the year to capture seasonal effects of eCO_2_ (*27*, *28*), following 6 years of eCO_2_ enrichment. Our objective was to investigate how higher N demands are being met and assess the potential for future N limitation by estimating soil N supplies and losses. We hypothesized that (i) N mineralization and N fixation will be up-regulated under eCO_2_ to sustain a higher tree N demand (*8*), and (ii) this up-regulation will follow seasonal patterns of higher soil C availability, leading to (iii) similar or lower N losses by coupled nitrification and denitrification processes (N_2_O and N_2_ gases) resulting from higher plant N uptake (*26*). To test our hypotheses, we assessed rates of belowground N processes in situ using ^15^N tracing incubations in intact soil cores to preserve the influence of roots. Gross ammonification, nitrification, N_2_O emissions, and BNF were measured in spring, summer, and autumn, and net mineralization was measured monthly for 1 year to quantify forest N fluxes.

## RESULTS AND DISCUSSION

### Enhanced N mineralization under enhanced tree N uptake

**Fig. 1.**
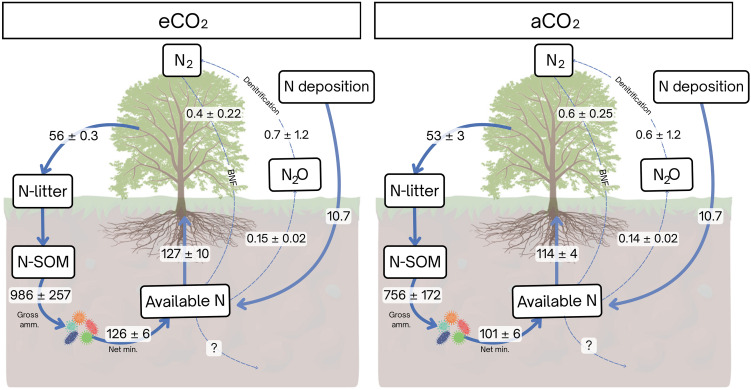
N fluxes in a temperate oak forest under aCO_2_ and eCO_2_. Schematic diagram of the N fluxes for the year 2022 based on measurements of N fluxes in the organic soil horizon (mean depth of 7.5 cm on average). All fluxes are expressed in kg N ha^−1^ year^−1^. N mineralization, BNF, and gaseous N losses were converted to kg ha^−1^ year^−1^ on the basis of the mean organic layer depth of 7.5 cm and the mean bulk density of 0.45 g cm^−3^. N atmospheric deposition data at the site were measured by analyzing the concentration in reduced and oxidized N collected monthly in 2022 in dry and wet collectors outside the forest. Note: N_2_O data are based on the current study, but there is evidence of reduced N_2_O emissions under eCO_2_ from in situ measurements (*51*).

**Fig. 2.**
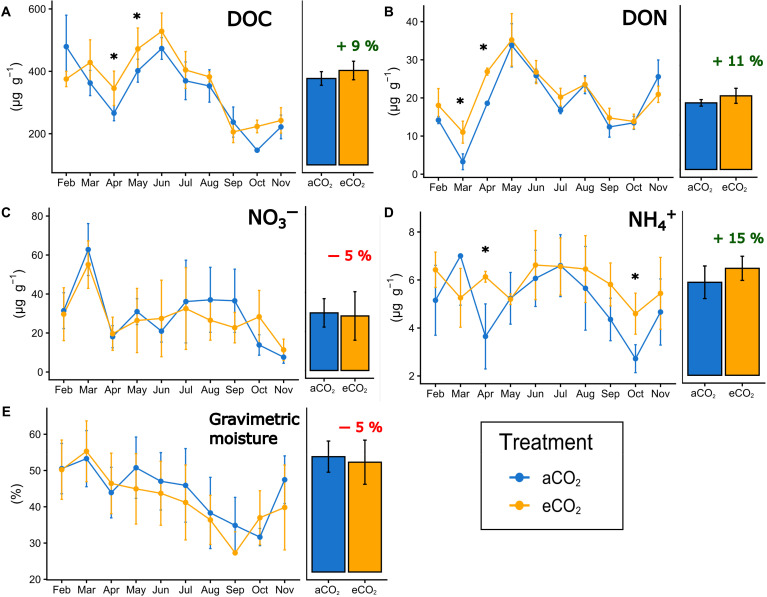
Soil chemical and moisture properties under aCO_2_ and eCO_2_. (**A**) DOC, (**B**) dissolved organic N (DON), (**C**) NO_3_^−^, (**D**) NH_4_^+^, and (**E**) soil gravimetric moisture measured monthly in the organic horizon from February to November 2022. Bar charts show the annual mean for each variable, and the percentages indicate the total positive or negative eCO_2_ effect over the study period. Error bars represent the standard error for *n* = 3; significant differences between treatments are represented by an asterisk over the bar plots.

**Fig. 3.**
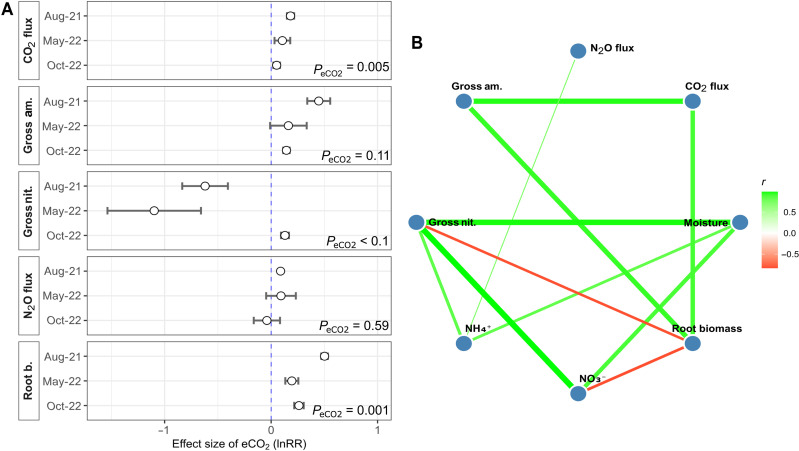
Effects of eCO_2_ on main soil processes and root biomass. (**A**) Response ratio of soil CO_2_ flux, gross ammonification (Gross am.), gross nitrification (Gross nit.), soil N_2_O flux, and fine root biomass (Root b.) to eCO_2_ with the *P* value of the eCO_2_ effect (*P*_eCO2_) shown. Error bars represent pooled variance (V) associated with the response ratios. (**B**) Correlation network based on Pearson correlation coefficients (threshold = 0.7), summarizing relationships among the latter variables, soil N content, and soil moisture across three sampling campaigns (August 2021, May 2022, and October 2022).

Our integrated N flux measurements revealed that after 6 years of CO_2_ enrichment, higher N demand by trees was sustained by higher net N mineralization, supporting our first hypothesis. Net N mineralization delivered an extra 26 kg N ha^−1^ year^−1^ of available mineral N (*P* < 0.05) for an extra 13 kg N ha^−1^ year^−1^ being taken up by the trees under eCO_2_ (Fig. 1). This resulted in higher ammonium (NH_4_^+^) availability in most months (+15%; *P* < 0.05; table S1), while nitrate (NO_3_^−^) availability was unaffected (Fig. 2). Net N mineralization matched tree N uptake under eCO_2_ (126 kg N mineralized for 127 kg N taken-up ha^−1^ year^−1^), underscoring its significance as a key N process for tree nutrition (*29*, *30*). In contrast, under ambient CO_2_ (aCO_2_), net N mineralization was lower than tree N uptake, suggesting that deposited Nr plays a role in meeting the tree N demand, possibly via direct canopy uptake (Fig. 1) (*31*). In situ gross ammonification rates were also higher under eCO_2_ over the three seasons measured (+30%; *P* = 0.11; Fig. 3A), and the microbial immobilization-to-ammonification ratio remained stable (∼87%; fig. S1), consistent with the higher microbial biomass N (*32*). Gross ammonification rates increased with soil respiration (+26%; *P* < 0.05) and fine root biomass (+39%; *P* < 0.05) measured in the intact cores during ^15^N tracing incubations (Fig. 3A) and were accompanied by an 11% increase in dissolved organic C (DOC; Fig. 2). These results suggest that N mining (mineralization of SOM-N) is stimulated in soils via greater root exploration (*32*) and increased C availability in soils (*33*) and likely facilitated by the presence of ectomycorrhizal (ECM) fungi (*34*). This is also supported by the weakened relationship between soil moisture and both soil respiration and ammonification, indicating that these processes became less moisture-driven under eCO_2_ (fig. S2). Instead, the positive relationship between ammonification and soil respiration suggests strongly coupled C and N mineralization under eCO_2_ resulting from C and N stoichiometry in SOM (*35*). Hence, increased N mineralization led to estimated extra soil C losses of ∼330 kg C ha^−1^ year^−1^ on the basis of the respiration rates in the incubated cores. Such losses match the extra C released by exudation under eCO_2_ [estimated at 350 kg C ha^−1^ year^−1^ in 2022 (*36*)], suggesting that the extra C from exudates is rapidly respired or that an equivalent amount is mineralized from SOM. The potential increase in SOM turnover aligns with biogeochemical models forecasting a trade-off between belowground and aboveground C resulting from nutrient mining (*19*). Furthermore, contrary to our first hypothesis, new N from BNF remained extremely low at site (0.5 kg N ha^−1^ year^−1^ on average; table S3) and was not up-regulated by higher C availability under eCO_2_. It is unlikely that Nr deposition saturated the forest with N, as atmospheric Nr deposition at the site (10.7 kg N ha^−1^ year^−1^ in 2022; Fig. 1) was moderate (*37*) and the soil C:N ratio remained high (∼18; fig. S3). Under ambient conditions, the amount of N sequestered in woody biomass (∼12 kg N ha^−1^ year^−1^) approximately matched Nr deposition. By contrast, under eCO_2_, N sequestration increased to ∼17 kg N ha^−1^ year^−1^ (*6*)—about 5 kg N ha^−1^ year^−1^ more than supplied by deposition—indicating that this additional N originates from internal cycling. Overall, our results suggest that an up-regulation of N mineralization from SOM sustained tree growth 6 years into CO_2_ enrichment. However, this up-regulation may not be sustained over time (*38*) as it will eventually deplete SOM-N stocks, as N return via leaf litter did not increase (Fig. 1). Although it would take decades to exhaust the total SOM-N stock (∼330 kg N ha^−1^), the pool of readily accessible SOM-N may decline much faster in this high C:N soil.

### Seasonal flexibility in N supplies to meet the N demand

**Fig. 4.**
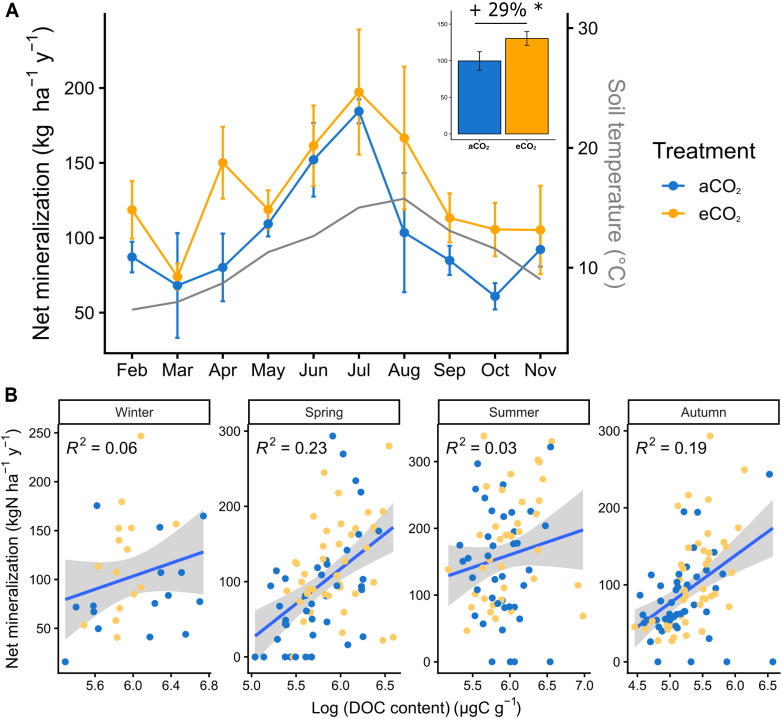
Net N mineralization in a mature temperate oak woodland under aCO_2_ and eCO_2_. (**A**) Monthly patterns of net N mineralization and soil temperature (gray line) from February to November 2022 and showing annual mean net N mineralization in the inserted bar plots and (**B**) regressions between net mineralization rates and log-transformed soil DOC content by season with the equations and *R*^2^ values. y, year. Error bars in (A) represent the standard error between treatments for *n* = 3 replicates.

High-frequency monitoring of N transformations revealed temporal variability in the magnitude of the eCO_2_ effect on net N mineralization (Fig. 4A). High net mineralization rates were closely linked to high soil DOC content, especially in spring and autumn (*R*^2^ = 0.20 and *R*^2^ = 0.17, respectively; Fig. 4B). In situ net mineralization was enhanced under eCO_2_ in April by 75% (*P* < 0.05; Fig. 4A), coinciding with the onset of tree leaf flushing, warmer soil, higher soil DOC content (+44%; Fig. 2), and higher root C exudation rates and fine root branching frequency (*36*). From May to July, net N mineralization was at its highest, reaching 185 kg ha^−1^ year^−1^, which was associated with high soil temperatures, but eCO_2_ had little effect (+9%; Fig. 4). The eCO_2_ effect on net mineralization may have been constrained by water availability, as the drought recorded at the study site in summer 2022 (*39*) was exacerbated under eCO_2_ (−10% gravimetric moisture; figs. S4 and S5) despite potential water savings resulting from higher water-use efficiency under eCO_2_ (*40*). This is in accordance with long-term data suggesting a trend toward drier soils under trees growing in eCO_2_ (*41*) because greater root biomass increases soil water uptake, while thicker leaves (*7*) increase canopy water storage and evapotranspiration capacity (*42*, *43*). Consequently, lower soil water content in eCO_2_ arrays during the dry summer probably limited net mineralization (*44*). From August to October, net mineralization was again enhanced under eCO_2_ (+50% relative to aCO_2_), coinciding with the tree nutrient resorption period (*45*), fresh litter decomposition, and higher ECM production rates at the site (*36*). Therefore, the seasonal effect of eCO_2_ on mineralization mirrors the seasonal effect on tree C allocation to root exudation and ECM production (*36*). Together, these findings suggest that trees under eCO_2_ increase specific C belowground fluxes to stimulate N mining when additional N is required for enhanced photosynthesis (*8*).

### Faster-yet-tighter N cycle under eCO_2_

Enhanced N mining under eCO_2_ did not translate to higher N losses, supporting our third hypothesis. Gross nitrification rates were unaffected in autumn but were two times slower in spring and summer (*P* < 0.1) despite higher DOC and DON (dissolved organic N) availability, which typically stimulates nitrification in acidic forest soils (*46*). This seasonal effect may reflect an intense competition for NH_4_^+^ during the growing season, reducing nitrification. This is in accordance with the observed seasonal effects of eCO_2_ on N_2_O fluxes at Duke FACE (i.e., lower fluxes than aCO_2_ in summer and higher fluxes than aCO_2_ in winter) (*25*). Furthermore, NO_3_^−^ content in the intact cores declined with increasing root biomass and with increasing gross ammonification, leading to decoupling between NH_4_^+^ and NO_3_^−^ pools under eCO_2_ (Fig. 3 and fig. S2). Thus, higher N uptake likely reduces gross nitrification by intensifying the competition for NH_4_^+^ as observed at the microscale of the rhizosphere (*32*, *33*) and in some other eCO_2_ experiments in contrasting ecosystems (*26*, *47*). While NH_4_^+^ uptake is the chief mechanism, higher root biomass may further diminish nitrification microsites and enhance the secretion of inhibitory compounds (Fig. 3B) (*48*–*50*). Discrete N_2_O measurements in intact soil cores showed no measurable effect of eCO_2_ against natural variability (Fig. 3A). However, continuous in situ measurements from 2020 to 2022 at BIFoR-FACE detected a 74% reduction in total N_2_O emissions (*51*). Conversely, laboratory measurements on root-free soils found higher potential N_2_O emissions in the bulk soil at site in 2022 (*P* < 0.05; fig. S6) and across previous years (*13*). These contrasting responses suggest that the root presence mitigates N_2_O emissions as part of a N conservation strategy by trees under eCO_2_ (*52*). Greater N conservation under eCO_2_ is also indicated by the higher C:N ratio of soil leachates (fig. S7) and root exudates (*36*), as well as an increase in N leaf resorption before leaf fall (*53*)*.* Furthermore, the higher C:N ratio observed in soil leachates (fig. S7) indicates reduced aqueous N losses under eCO_2_. Together, these findings point to a faster (i.e., higher N mineralization) yet tighter N cycle facilitated by plant-soil interactions and ecosystem’s ability to conserve N. Similar patterns have been reported previously using modeling and other approaches (*54*–*56*), suggesting that enhanced N retention may delay N limitation under future climate scenarios. Reduced N losses may partially compensate the extra 5 kg of N locked in woody biomass per year—although the magnitude of reduction remains uncertain. Hence, tightly coupled N cycling between microbes and plants suggests that the forest may be operating near a tipping point, where a decline in external N inputs (e.g., N deposition) could constrain plant responses to eCO_2_ despite enhanced N conservation (*57*–*59*).

By establishing a forest N budget after 6 years of CO_2_ enrichment, we demonstrate that greater net primary productivity was sustained through enhanced N mineralization and N conservation at the ecosystem scale. Linking the seasonal patterns of N mineralization observed here to previous work on tree C allocation indicates that trees under eCO_2_ increase belowground C fluxes to stimulate N mining from SOM according to seasonal demand. Future work should assess for how long faster and tighter N cycling under eCO_2_ can alleviate progressive N limitation at decadal timescales. Given the role of Nr deposition in delaying progressive N limitation, projecting future Nr deposition trajectories will be crucial to determine whether and when N limitation will constrain the CO_2_ fertilization effect. Hence, climate policies should consider how both C and N emission pathways can affect the 21st-century C uptake of temperate forests.

## MATERIALS AND METHODS

### Experimental setup

The study was performed at the BIFoR-FACE facility in a mature deciduous forest in Staffordshire (52°48′3.6″N, 2°18′0″W), UK. The forest is dominated by 180-year-old English oak (*Q. robur* L.) reaching up to 25 m in height and comprising 92% of total basal area. The understory includes hazel (*Corylus avellana* L.), sycamore (*Acer pseudoplatanus*), holly (*Ilex aquifolium* L.), and hawthorn (*Crataegus monogyna* L.), and the ground layer is covered by fern (*Dryopteris* sp.), bramble (*Rubus fruticosus* agg.), and bluebell (*Hyacinthoides* sp.). The soil is classified as a Ortic Luvisol (*60*) with a sandy loam structure and a pH around 4.5 (*61*). The organic layer (O) is 6 to 9 cm deep and features a high fine root density (1 mg cm^−3^), a high C content (10%), and a low bulk density (0.45 g cm^−3^). Mineral layers (A and B) featured mostly coarse roots with a medium packing density and a soil pH around 5 (*61*). The mean annual air temperature at the site is 10.8°C, and the mean annual precipitation is 1152 mm.

The BIFoR-FACE facility is composed of six infrastructure arrays (30 m in diameter); three are fumigated with a target concentration of +150 parts per million (ppm) of CO_2_ above ambient (eCO_2_) and three with ambient air (aCO_2_; fig. S8). In 2022, the average CO_2_ concentrations were 573 ppm in the eCO_2_ plots and 424 ppm in the aCO_2_ plots. Fumigation started in April 2017 and operated in daylight hours from budburst (∼1st April) to leaf fall (∼1st November). The performance of the system has been reported by Hart *et al.* (*60*). For a detailed description of the site, see the work of Hart *et al.* (*60*), and for the general analytical pipeline, see the work of MacKenzie *et al.* (*41*).

In each array, three sampling plots (0.5 m by 0.5 m) were established, each at ∼1-m distance from three different oak trees. For the present study, five pseudoreplicate sampling locations were randomly selected among the three sampling plots. At each sample collection, the leaf litter was first removed (Oi), and the remaining organic layer was sampled (Oe + Oa). The sampling depth varied from 6 to 9 cm depending on the depth of the Oe + Oa horizon (henceforth “O horizon”).

### Monthly measured variables

#### Soil nutrient pools

Soil nutrient concentrations in the organic layer were measured monthly from February to November 2022. Soil NH_4_^+^ and NO_3_^−^ concentrations were measured by 0.5 M potassium sulfate [K_2_SO_4_; soil:extractant ratio of 1:5 (w/v)] extraction and continuous flow colorimetric analysis (Skalar SA 3000 analyzer, The Netherlands). The soil concentrations of DOC and total dissolved N were measured after extraction with 0.5 M K_2_SO_4_, and extracts were analyzed on a TOC/TN analyzer (Multi N/C 2100, Analytik Jena, Germany). Gravimetric moisture was measured by drying 5 g of soil at 105°C for 48 hours. Additional information on analyses can be found in the work of Rumeau *et al.* (*32*).

#### Net N mineralization

Monthly net N mineralization was measured in the organic layer from February to November 2022. Each month, soil samples (O-horizon) were collected from the five sampling locations per array, sieved to 2 mm and homogenized on site. A subsample (∼10 g) was incubated for 28 days in a polyethylene bag buried in situ under the leaf litter. The polyethylene bag was water-tight but air-porous to facilitate gas exchange. Net mineralization was assessed by comparing the inorganic N content (NH_4_^+^ + NO_3_^−^) measured upon sample collection with values measured in the incubated samples after a 28-day incubation period (*62*). Given that the incubated soil was isolated from roots, we only estimated total net N mineralization, as the absence of root competition with nitrifying bacteria can lead to inaccurate estimates of net ammonification and nitrification (*63*). The annual estimate was calculated and converted to kg ha^−1^ year^−1^ on the basis of a mean organic layer depth of 7.5 cm and a mean bulk density of 0.45 g cm^−3^.

### Seasonally measured variables

#### In situ gross ammonification, nitrification, and N_2_O emissions

Gross rates of N ammonification and nitrification were measured in August 2021, May 2022, and October 2022 using the ^15^N isotopic pool dilution method adapted for field incubation of intact soil core (*64*) to maintain the influence of roots. We further adapted the method to include the rate of N_2_O emissions from nitrification and denitrification using the ^15^N gas flux method (*65*). The full methodology is described in detail below.

Within a week before ^15^N labeling, soil NH_4_^+^ and NO_3_^−^ content was measured in each pseudoreplicate location to target ∼20% enrichment of N pool size during labeling. A range of labeling solutions were prepared to simulate the different soil inorganic N concentrations, and the solutions were flushed with oxygen-free N_2_ to avoid introducing oxygen to the soils during labeling. In each pseudoreplicate location, a pair of intact O-horizon soil cores (containing cut roots; *T* = 24–hour samples) was sampled using a soil corer (5 cm in diameter) with a slide hammer, and a pair of disturbed soil samples (*T* = 0–hour samples) was sampled to the same depth with a hand auger and directly transferred in a cool box for transfer to the lab. The intact soil cores were labeled with K^15^NO_3_ or with ^15^NH_4_Cl by multiple injections with a long needle into the soil to ensure homogeneous labeling and were then incubated in situ for 24 hours. After 24-hour incubation, the samples were mixed by hand, and 15 g of subsample was extracted with 1 M KCl (1:5, w/v). A subsample of the extracts was analyzed by continuous flow colorimetry analysis (Skalar SA 3000 analyzer, The Netherlands) for inorganic N content. The rest of the extract sample (∼50 ml) was used to extract the ^15^N content in the NH_4_^+^ and NO_3_^−^ pools using a two-step gas diffusion procedure (*13*, *66*). Briefly, NH_4_^+^ and NO_3_^−^ pools were diffused onto an acidified filter for 6 days, and the filters were analyzed on an elemental analyzer-isotope ratio mass spectrometer (EA-IRMS; Elementar Isoprime Precision; Elementar Analysensysteme GmbH, Hanau, Germany) (*13*).

To assess fine root biomass, roots from the intact soil cores were washed with deionized water and dried at 65°C, and the fine root biomass (diameter ≤1 mm) was quantified per soil volume. To ensure that ^15^N uptake by cut roots did not affect calculations of gross N transformation rates (*67*), a subset of root samples (three per array) was pulverized and analyzed for ^15^N content, as described above. The root N uptake was <0.2 μg g day^−1^ and was therefore considered negligible compared with N transformation rates.

To determine gaseous losses, gas samples were taken from the soil cores in the field at *T* = 0 hours and after 24 hours of incubation with a syringe through the septum of the core lid and transferred to a pre-evacuated 12-ml gas Exetainer vial (Labco, Ceredigion, UK). The gas samples were analyzed for total CO_2_ and N_2_O concentrations, as described by Rumeau *et al.* (*32*). Gross N ammonification and nitrification rates were calculated using the equations developed by Kirkham and Bartholomew (*68*), and N_2_O emissions from nitrification and denitrification were calculated as described by Sgouridis *et al.* (*13*). All formulas are detailed in the Supplementary Text.

As N_2_ flux from denitrification was not detectable in intact soil cores under a natural atmosphere, lab incubation was conducted under an artificial atmosphere and controlled moisture to measure potential N_2_ losses via denitrification using the ^15^N gas flux method (*68*). Briefly, soil samples were placed in air-tight 100-ml serum bottles and labeled with K^15^NO_3_ targeting simultaneous 25% enrichment of the NO_3_^−^ pool and 65% soil gravimetric moisture. The serum bottle atmosphere was replaced by an artificial atmosphere depleted in N_2_ (5% N_2_, 20% O_2_, 75% He, and 0.11 ppm of N_2_O), and an initial gas sample was taken and transferred into a 12-ml gas Exetainer vial. Gas samples were taken from the serum bottles after 6 and 24 hours of incubation and were analyzed on an EA-IRMS coupled with a trace-gas preconcentrator inlet. The ratio of N_2_O/(N_2_O + N_2_) was calculated using the equations described by Micucci *et al.* (*65*).

#### Biological N fixation (BNF)

In situ rates of BNF were assessed by the ^15^N assimilation method (*69*). O-horizon soil samples were collected from the same sampling locations as previously in June 2021, September 2021, and April 2022. In each array, the five soil cores collected were bulked to make one composite sample. The samples were sieved (<2 mm) on site, and eight 10-g subsamples were weighed into 50-ml dark air-tight serum bottles. Five of the eight subsamples were incubated with an atmosphere enriched in ^15^N_2_ at 20% by replacing 5 ml of the headspace with ^15^N_2_ gas (98 at. % ^15^N, Sigma-Aldrich), and three of the subsamples were incubated with ambient air (controls). Negative standards were made of autoclaved soil and followed the same procedure. All samples were incubated in situ for 24 hours before aerating the samples to stop the incubation. Samples were dried at 70°C for 72 hours, pulverized, and analyzed on an EA-IRMS. The analytical precision limit of the EA-IRMS (limit of detection) was determined by repeated measurements of bulk soil natural abundance of ^15^N. The formula for BNF rate calculation is detailed in the Supplementary Text.

#### Tree N uptake and litter fall inputs

N uptake by trees was calculated on the basis of net primary productivity calculations in 2022 (*6*) and on N pools in different tree compartments: wood (including coarse roots and stems), fine roots, and leaves (see table S3). Oak wood cores were extracted from five trees per treatment, and latewood formed in 2022 was analyzed for N content. Fine root N content was estimated as the average N concentration in fine roots (<2 mm in diameter), sampled from the top three soil horizons (O, A, and B) at three locations per array in July—the period of maximum N content. The green leaf N concentration was also measured in July to capture peak foliar N content. For oak, green leaves were collected at three canopy levels from two trees per array; for sycamore and hazel, one tree per array was sampled. Leaf litter N content was analyzed to estimate the amount of N reabsorbed from senescing leaves in 2021. Six litter traps were installed in each array, and litter was periodically collected and sorted by species to estimate the amount of N reabsorbed from senescing leaves in 2021. N inputs via litter fall in 2021 were estimated from the dry mass and N concentrations of litter collected in the litter traps.

### Statistical analyses

Statistical analyses were carried out in R (version 4.1.0) using Rstudio software (version 1.4.1103) (*70*). The effects of eCO_2_ treatments on individual variables were tested with linear mixed-effect models [“lmer” function in the lme4 package (*71*)], with treatment as a fixed effect, sampling time and array as random effects, and soil moisture as a covariate to adjust for the soil moisture effect. Because of the high variability and low replication in large-scale FACE experiments (*n* = 3), we considered a *P* value between 0.1 and 0.05 as a biologically relevant effect and a *P* < 0.05 as statistically significant. When assumptions of normality and homogeneity of variance were not met, log transformations were performed to meet model assumptions. To estimate the eCO_2_ effect gross ammonification, nitrification, CO_2_ and N_2_O flux, and fine root biomass, we calculated the natural log of the response ratio (RR) as an effect size and its corresponding pooled variance (V) (*72*) between the eCO_2_ and aCO_2_ treatments. Correlation networks were built using the Pearson correlation index with a threshold defined at 0.7 to study C and N coupling.
