# Supplementary material for: Faster-and-tighter nitrogen cycle supports mature forest productivity under elevated CO2
Source: Sci Adv. 2026 Jul 29;12(31):eaed2732. doi: 10.1126/sciadv.aed2732 (PMC13445031; doi:10.1126/sciadv.aed2732)
Supplement: Supplementary file 1 [file sciadv.aed2732_sm.pdf]

Science Advances
AAAS

Supplementary Materials for

Faster-and-tighter nitrogen cycle supports mature forest productivity under elevated CO_2

Manon Rumeau *et al.*

Corresponding author: Manon Rumeau, manon.rumeau@univ-pau.fr; Sami Ullah, s.ullah@bham.ac.uk

Sci. Adv. **12**, eaed2732 (2026)
DOI: 10.1126/sciadv.aed2732

This PDF file includes:

Tables S1 to S5
Supplementary Text
Figs. S1 to S8

Table S1: Effect of eCO_2 and period and their interactions shown as a model F statistic and *P* value. Bod typing indicate a significative effect (*P* <0.05), and a dash line indicate the switch between monthly measured variables (DF = 9) to seasonally measured variables (DF = 2).

Factors	eCO_2		Period		eCO_2 x Period	
DF	1		9 (or 2)		9 (or 2)	
Analysis of variance	F statistic	*P*	F statistic	*P*	F statistic	*P*
Gravimetric moisture	0.04	0.84	9.21	**<0.0001*** **	0.89	0.53
DOC	2.73	0.17	18.49	**<0.0001*** **	1.76	**0.03***
DON	7.43	**0.006** **	8.40	**<0.0001*** **	0.82	0.82
NH_4^+	4.04	0.11	5.96	**0.005** **	2.87	**0.008** **
NO_3^-	0.11	0.75	15.28	**<0.0001*** **	1.18	0.30
Net mineralization	12.3	**0.02***	8.22	**<0.0001*** **	0.77	0.63
Gross ammonification	2.51	0.11	3.0	**0.05***	0.6	0.54
Gross nitrification	2.84	**0.09**	1.84	0.16	1.01	0.36
NH_4^+ immobilization	3.08	**0.08**	0.09	0.91	1.7	0.19
NO_3^- immobilization	1.10	0.35	4.68	**0.01***	3.03	**0.05***
Soil CO_2 flux	12.3	**0.0005*** **	78.3	**<0.0001*** **	3.9	**0.009** **
Root biomass	11.1	**0.001** **	1.2	0.3	0.9	0.40
N_2O emissions	0.27	0.59	15.5	**<0.0001*** **	0.12	0.87
N_2O per nitrification	1.6	0.20	1.1	0.33	0.12	0.88
N_2O per denitrification	0.004	0.98	2.7	**0.07**	0.7	0.49

Table S2: Nitrogen fixation in the six arrays at three time points (June and September 2021 and April 2022) in nmol g^{-1} d^{-1}. Different letters indicate significant difference between the six arrays at the specific period. LOD = limit of detection set at 0.14 nmol g^{-1} d^{-1} from repeated IRMS analysis. Data are means + SE (n=5).

	**eCO_2**			**aCO_2**		
	A1	A4	A6	A3	A2	A5
**June 2021**	<LOD a	0.19 ± 0.04 a	0.71 ± 0.56 a	<LOD a	0.31 ± 0.16 a	0.63 ± 0.33 a
**September 2021**	<LOD a	<LOD a	<LOD a	0.19 ± 0.04 a	0.57 ± 0.42 a	0.28 ± 0.13 a
**April 2022**	<LOD b	0.22 ± 0.06 b	0.37 ± 0.15 b	<LOD b	0.17 ± 0.02 b	0.66 ± 0.17 a

Table S3: Mean N concentrations (%) in the different tree compartments (wood, fine roots, leaves and litter) with mean value of N uptake in kg ha^{-1} calculated in each array for 2022 based on these N pools and the net primary productivity calculation that year.

	eCO_2			aCO_2		
	A1	A4	A6	A3	A2	A5
N in wood (%)	0.286	0.286	0.286	0.283	0.283	0.283
N in fine roots (%)	1.00	1.29	1.31	1.15	1.13	1.20
N in oak leaves (%)	3.01	2.56	2.62	2.60	2.99	2.73
N in sycamore leaves (%)	2.63	2.88	2.54	2.44	2.53	2.21
N in hazel leaves (%)	2.57	2.39	2.19	2.29	2.17	2.45
N in oak litter (%)	1.05	1.21	1.26	1.38	1.27	1.38
N in sycamore litter (%)	2.40	1.49	2.15	1.24	0	2.02
N in hazel litter (%)	1.29	1.02	1.63	1.28	1.14	1.50
**N uptake (kg.ha^{-1})**	**102.4**	**142.9**	**138.5**	**103.7**	**118.7**	**119.9**

Table S4: Mean and standard deviation of soil variables measured monthly in 2022 under eCO_2 and aCO_2.

treatment	Period	Net mineralization	NO_3^-	NH_4^+	DC	DON	moisture	temperature
		kg ha^{-1}	µg g^{-1}	µg g^{-1}	µg g^{-1}	µg g^{-1}	%	°C
**aCO_2**	Feb	87.1±46.7	31.4±27	5.1±2.7	479.4±218.9	14.2±7.8	50.5±14.9	6.5
	Mar	56.7±92	62.8±37.2	7±4	362.6±141.1	3.1±5.2	53.2±20.1	7.14
	Apr	80.1±69.4	18.1±15.3	3.6±2.1	266.1±70.4	18.6±5.9	43.9±13.5	8.71
	May	108.7±63.6	30.9±21.7	4.9±2.4	399.1±117.2	32.3±13.2	50.7±15.9	11.3
	Jun	148.6±71.8	20.9±19.6	6±2.8	473.2±140	25.8±6.3	47±18.5	12.64
	Jul	183.3±55.5	36.1±46.1	6.4±3.1	369.6±159	16.6±9.2	45.8±20.5	15.02
	Aug	102.3±98.4	37±38.1	5.4±3.4	353.1±119.6	22.9±6.3	38.2±19.9	15.76
	Sep	84.8±41.7	36.5±41.1	4.2±2.8	237.1±149.5	12.9±7.1	34.8±18.5	13.08
	Oct	60.9±31.6	13.8±15.7	2.6±1.6	146.9±45.3	13.8±5.6	31.6±9.1	11.6
	Nov	92.1±69.7	7.6±9.4	4.6±2.8	221.8±141.8	25.5±20.1	47.4±17.1	9.02
**eCO_2**	Feb	118.6±55.2	29.6±24.9	6.4±1.9	375.5±88.8	18.6±10.5	50.2±14.9	6.5
	Mar	70.3±47.1	55±23.5	5.2±2.1	428.4±132.3	9.5±8.9	55.2±15.4	7.14
	Apr	150.1±62.4	19.6±14	6.1±2.2	345.6±120.3	26.8±11.4	46.4±14.9	8.71
	May	118.7±57.4	26.4±30.6	5.1±1.7	472.1±151	36.7±21	44.9±16.9	11.3
	Jun	162±81.9	27.4±37.8	6.1±2.5	528.4±245.4	27.1±8.5	43.7±16.8	12.64
	Jul	194.4±78.7	32.5±38.7	6.4±2.7	404.3±137.5	20.6±9.1	41.1±19.3	15.02
	Aug	173±87.4	26.5±22.9	6.4±2.8	382.3±96.5	23.5±7.6	36.3±16.4	15.76
	Sep	113.2±62.5	22.7±15.9	5.7±2.5	208.4±85.4	14.6±5.9	27.2±11.7	13.08
	Oct	105.5±74.7	28.3±24.6	4.7±2.2	223.3±73.2	13.5±5.9	36.9±16.3	11.6

Nov	105.2±56.7		11.3±11.2	5.4±2.8	244.2±102.5	20.8±7	39.8±19.3		9.02

Table S5: Mean and standard deviation of N cycling rates, fine root biomass and CO2 flux measured in August 2021, May 2022 and October 2022 used for the calculation of response ratio in Figure 4A.

	August		May		October	
	aCO_2	eCO_2	aCO_2	eCO_2	aCO_2	eCO_2
Gross ammonification (μg g^{-1} d^{-1})	6.79±3.45	10.63±2.7	4.91±2.52	5.78±2.91	5.83±1.03	6.73±1.64
Gross nitrification (μg g^{-1} d^{-1})	10.3±4.95	5.54±3.55	6.47±2.81	2.16±2.29	3.51±1.1	3.99±0.44
Fine root biomass (mg cm^{-3})	0.79±0.101	1.31±0.365	0.82±0.165	1±0.379	0.96±0.144	1.25±0.419
CO2 flux (μg g^{-1} h^{-1})	1.24±0.38	1.49±0.08	0.59±0.25	0.65±0.12	0.49±0.05	0.51±0.14
N2O flux (μg g^{-1} h^{-1})	0.08±0.006	0.08±0.015	0.05±0.021	0.05±0.028	0.02±0.016	0.02±0.001

Supplementary Text: Calculation descriptions for N transformation rates using ^{15}N isotopic labelling methods.

Gross N transformations

Gross N fluxes were calculated using the following equations developed by Kirkham and Bartholomew (1954):

$$M\ (\mu g\ g^{-1}\ day^{-1}) = \frac{[NH_4^+]_0 - [NH_4^+]_t}{t} \times \frac{\log\left(\frac{APE_0}{APE_t}\right)}{\log\left(\frac{[NH_4^+]_0}{[NH_4^+]_t}\right)} \quad (1)$$

$$C\ (\mu g\ g^{-1}\ day^{-1}) = M - \frac{[NH_4^+]_t - [NH_4^+]_0}{t} \quad (2)$$

Where M= gross ammonification rate, C= NH_4^+ consumption rate, t = time elapsed, [NH_4^+] = NH_4^+ soil concentration (µg g^{-1}), APE_τ = ^{15}N atom percent excess of the NH_4^+ pool, τ.

Gross rates of nitrification and NO_3^- consumption were calculated by replacing NH_4^+ concentration by NO_3^- concentration, and APE in the NH_4^+ pool by the APE in the NO_3^- pool. NH_4^+ immobilization rate is equal to the difference between NH_4^+ consumption rate and gross nitrification. NO_3^- immobilisation rate is assumed equivalent to the gross NO_3^- consumption rate.

Total N_2O and CO_2 flux

Total N_2O emissions and basal respiration (i.e., CO_2 flux) were calculated using the following equation (CO_2 flux example):

$$CO_2\ flux\ (\mu gC\ g^{-1}\ h^{-1}) = \frac{([CO_2]_t - CO_2]_0) \times H}{m \times t} \quad (3)$$

Where $[CO_2]_\tau$ = concentration of CO_2 in µg C L^{-1} in the vial, H = headspace in L, m = mass of dry soil (g), t = time elapsed (h). N_2O flux is calculated in the same way by substituting the concentration in N_2O in µg N L^{-1} in the above equation.

N_2O from nitrification and denitrification

N2O production from nitrification and denitrification was calculated using the ^{15}N Gas Flux method. For each gas sample, isotopic ratio R29 (45/44) and R30(46/44) were calculated and corrected for oxygen isotope natural abundance. The coefficient ap (^{15}N enrichment of the nitrate or ammonium pool) was calculated, allowing the calculation of the coefficient d representing the fraction of produced N2O originating from the labelled N pool.

N2O production from nitrification (from NH_4^+ label) and denitrification (from NO_3^- label) was calculated as following and resulting rates were converted to ng N g^{-1} h^{-1}

$$N_2O\ from\ (de)nitrification = d\ x\ (N_2O_{tf} - N_2O_{t0}) \quad (4)$$

N_2 flux from denitrification

Denitrified N_2 was estimated using the ap coefficient from N2O isotopic data. The coefficient d’ was calculated as

$$d' = \frac{1 - R29\,(1-ap)^2 - 2ap\,(1-ap)}{R29\;(1-aa)^2 - 2aa\,(\,1-aa)} \qquad (5)$$

where aa is the ^{15}N abundance of atmospheric N_2.

Denitrified N_2 concentration was then determined as:

$$Denitrified\ N_2 = d\ x\ N_{2_{background}} \qquad (6)$$

where $N_{2_{background}}$ is the total N_2 concentration measured in the vials.

Biological N fixation

The BNF rate was calculated with Equation 7.

$$Y = \left(\frac{atom\%^{15}Nexcess}{100}\right) x \left(\frac{totalN\ x\ 10^9}{t\ x\ 28}\right) x \left(\frac{100}{\%^{15}N\ headspace}\right) \qquad (7)$$

Where Y is the amount of N fixed during the incubation (nmol N g^{-1} h^{-1}), $atom\%^{15}Nexcess$ is the difference of ^{15}N between enriched and control samples, $totalN$ is the total nitrogen content in the sample in %, t is the incubation time (24 h) and $\%^{15}N\ headspace$ is the amount of $^{15}N_2$ in the serum bottle (20%).

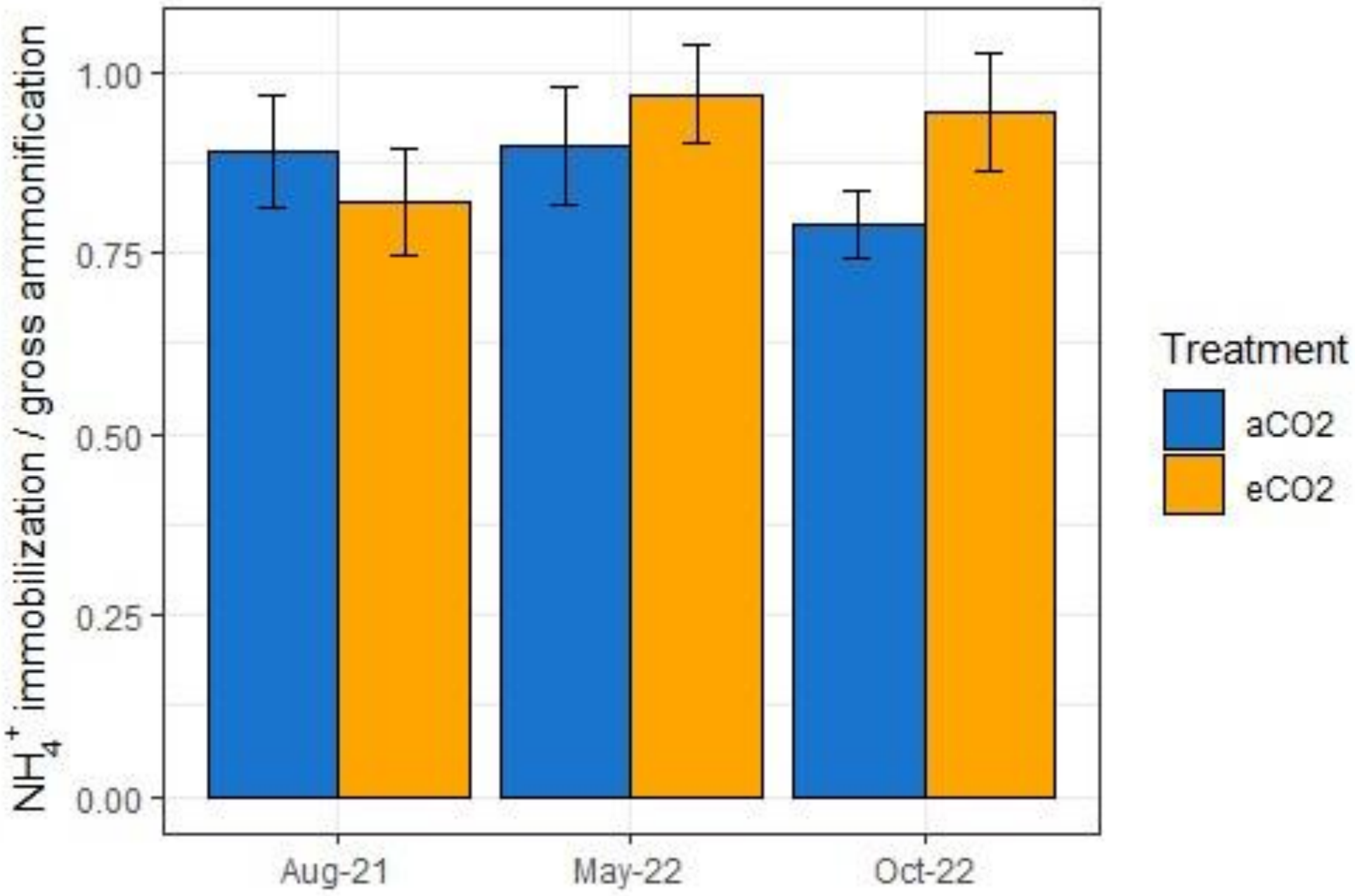

Fig. S1: Ratio of NH_4^+ immobilization over gross ammonification rates from *in-situ* ^{15}N incubation in August 2021, May 2022 and October 2022 under ambient (aCO_2; blue) and elevated CO_2 (eCO_2; orange). Data are means + SE (n=3).

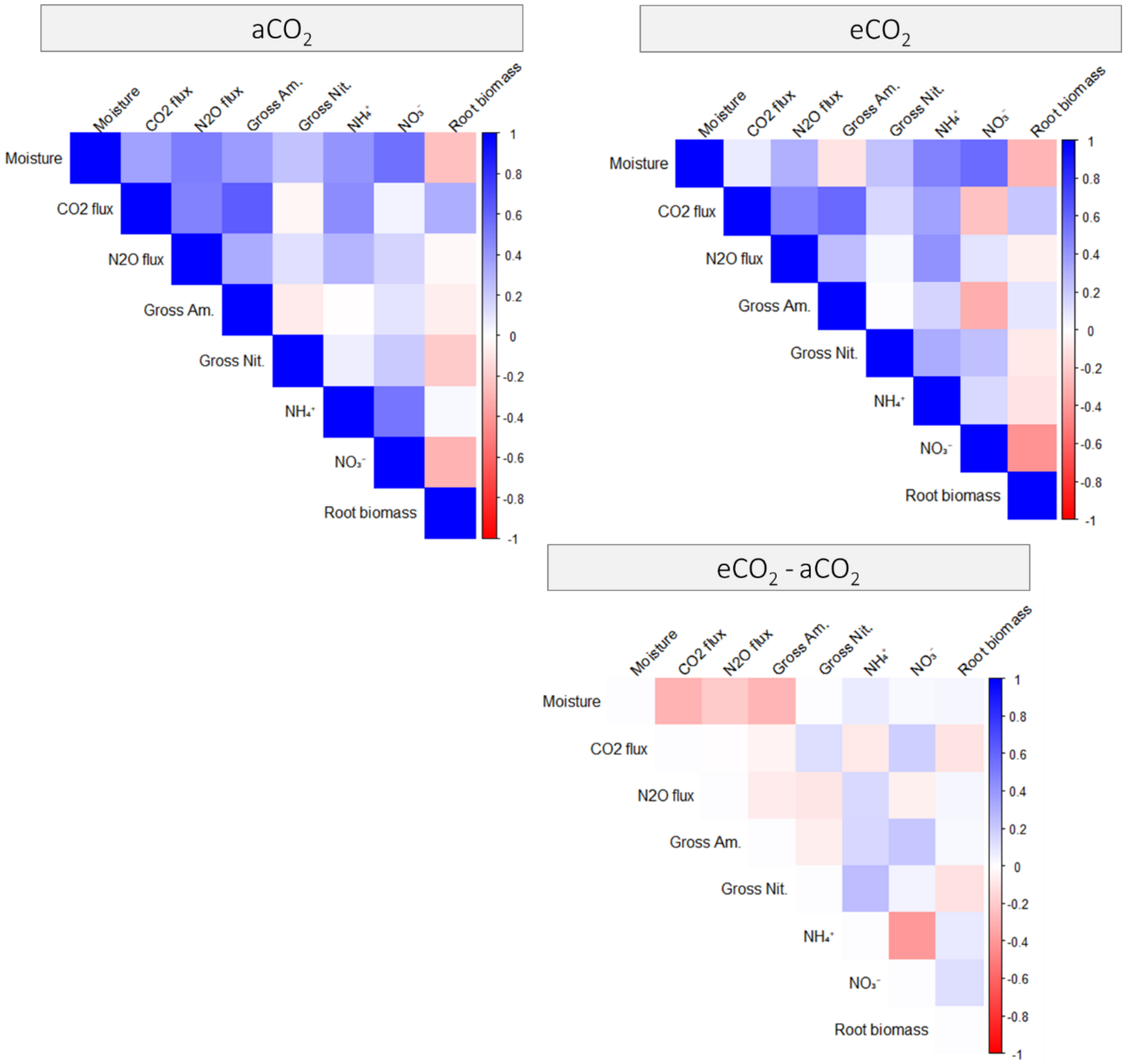

Fig. S2: Heatmap based on Pearson correlation coefficients showing correlation among variables measured across three sampling campaigns (August 2021, May, 2022 and October 2022) under aCO_2 and eCO_2 (top panels). The bottom panel (eCO_2 - aCO_2) displays the absolute difference in correlation strength, illustrating the coupling or decoupling of system-level processes in response to elevated CO_2.

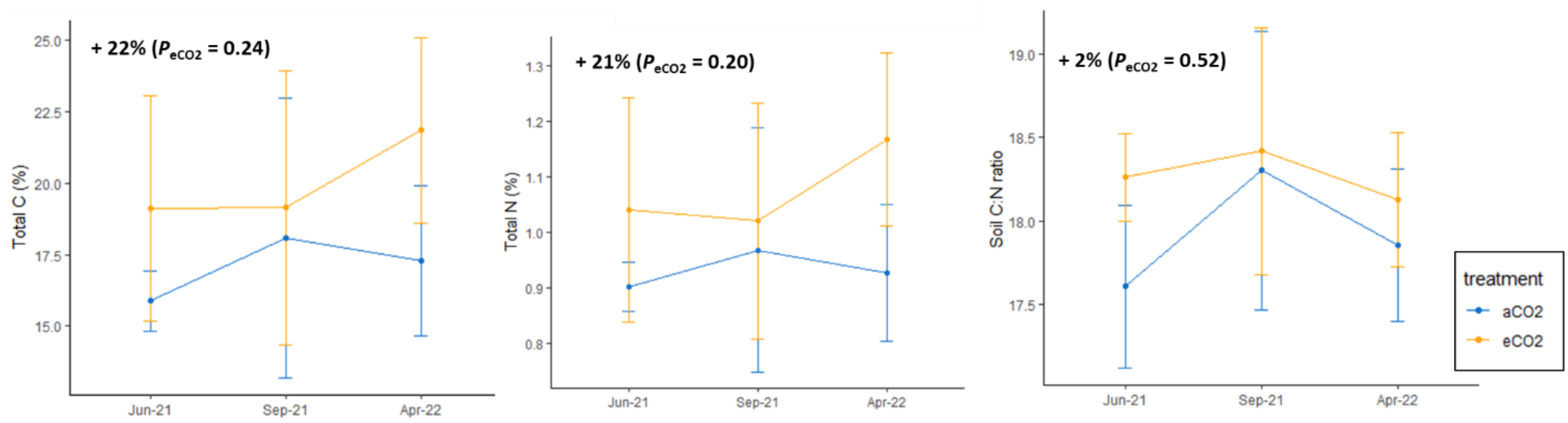

Fig. S3: Soil total C, N and C/N ratio at three sampling times (June 2021, September 2021, and April 2022) under ambient (aCO_2; blue) and elevated CO_2 (eCO_2; orange). Data are means + SE (n=3).

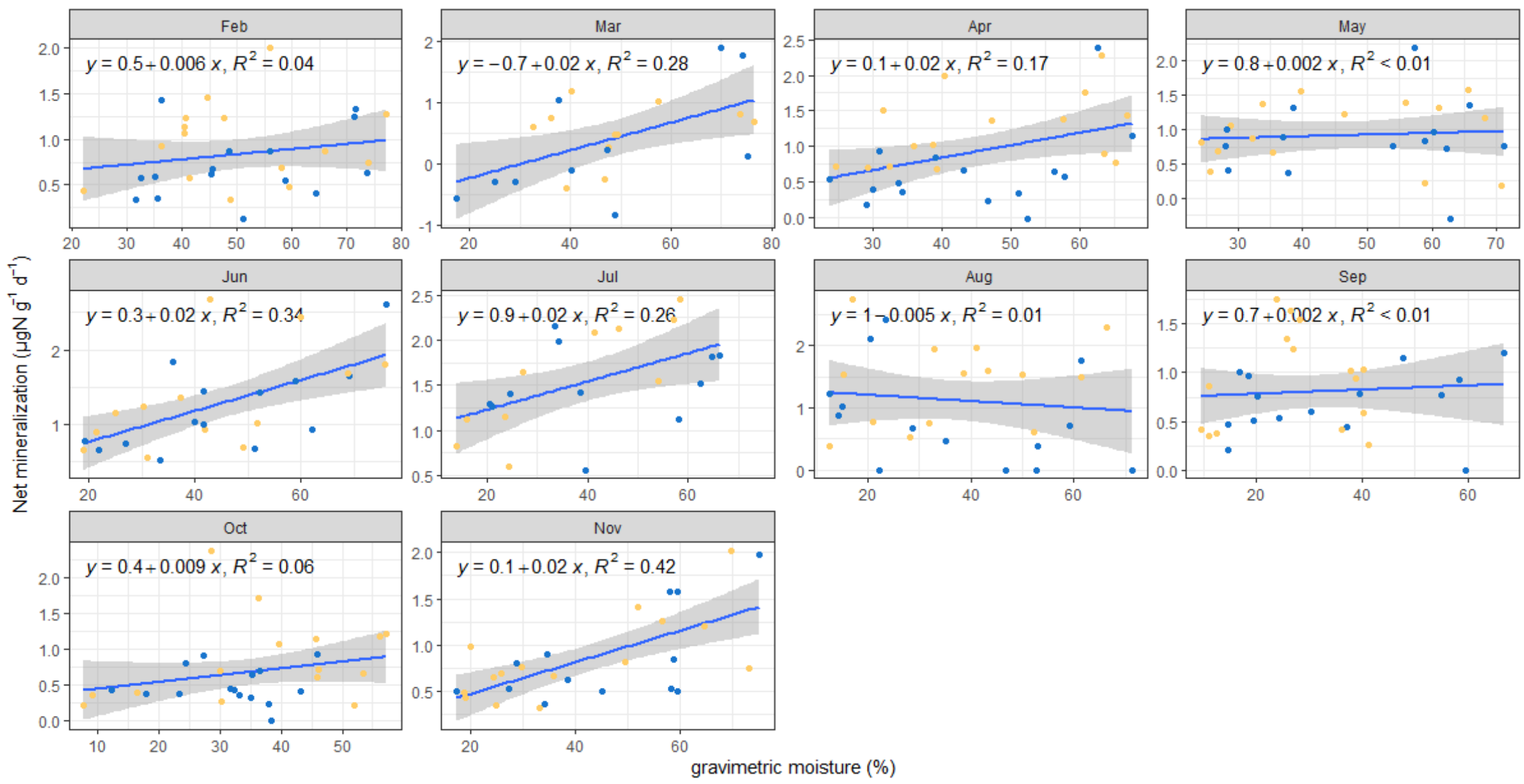

Fig. S4: Regressions between net mineralization rates and soil gravimetric moisture on a monthly basis from February 2022 to November 2022 (orange= eCO_2, blue = aCO_2) with the equations and R^2 values.

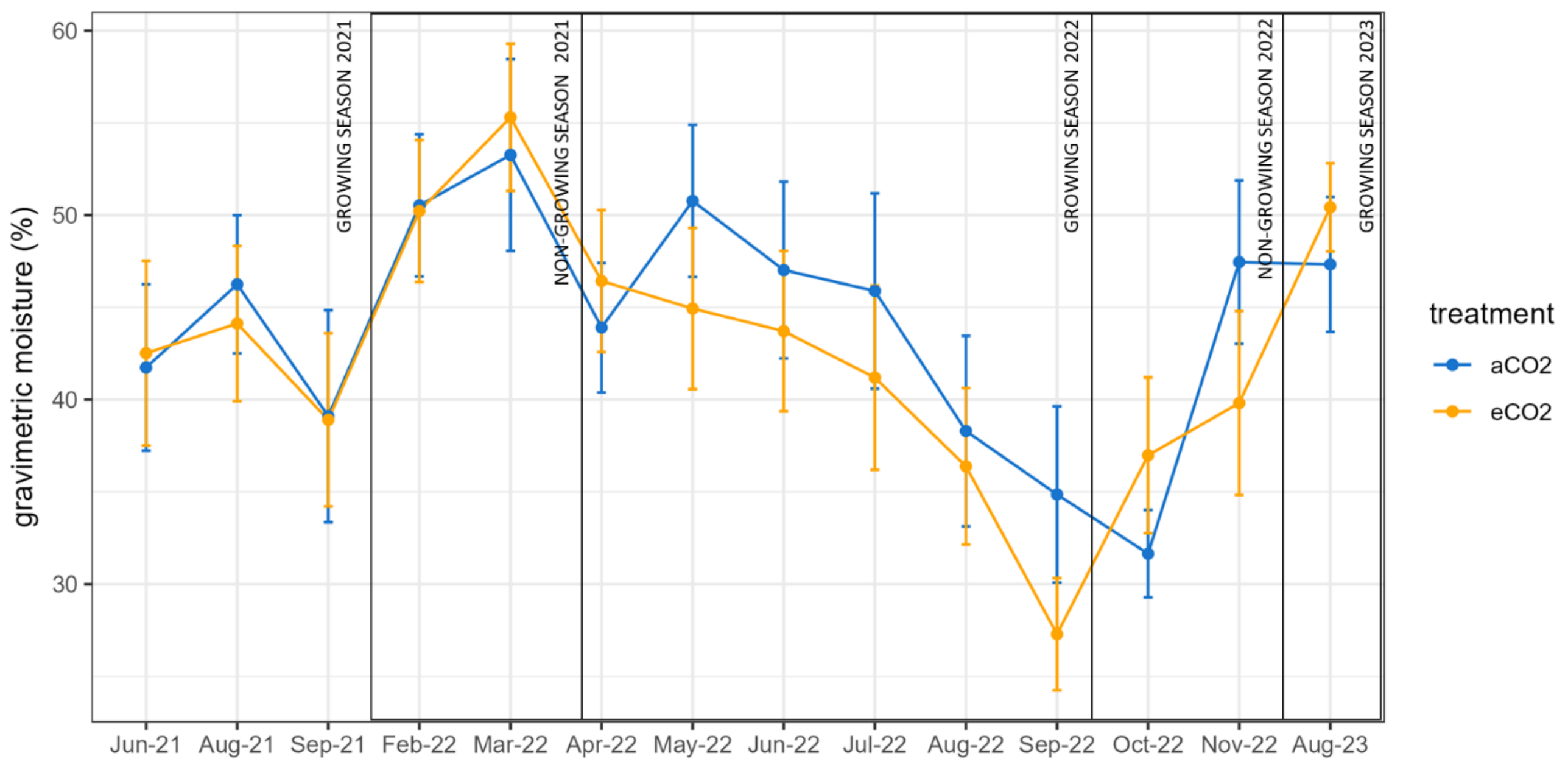

Fig. S5: Soil gravimetric moisture under eCO_2 (orange) and aCO_2 (blue) from June 2021 to August 2023. Error bars represent the standard error between replicates (n=3). Periods are separated between growing (April to October) and non-growing (November to March) seasons.

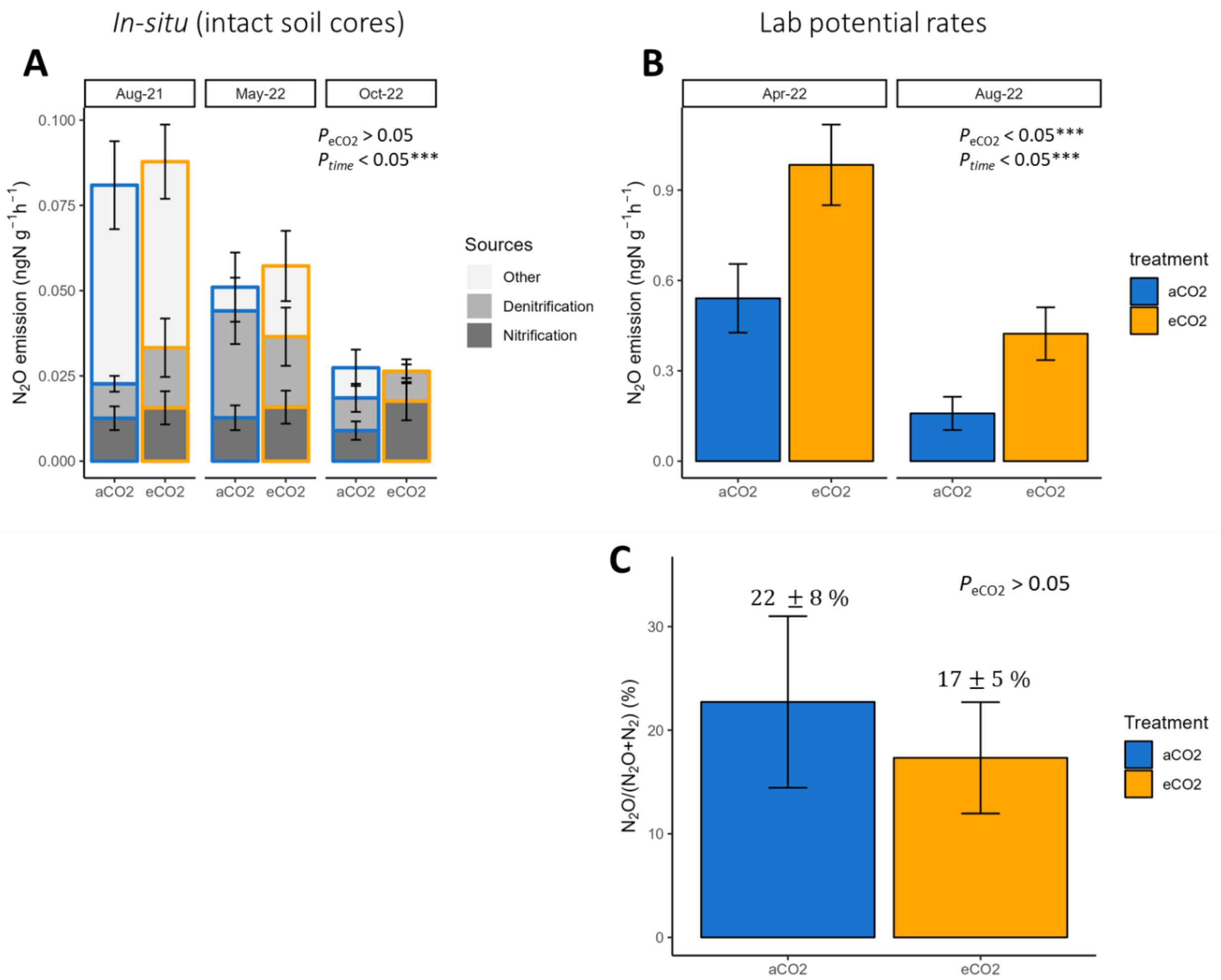

Fig. S6: A) Nitrous oxide (N_2O) emission with source partitioning (from nitrification, from denitrification or other sources) at the 3 sampling dates (August 2021, May 2022 and October 2022) under ambient (aCO_2; blue) and elevated CO_2 (eCO_2; orange). B) Potential total N_2O emissions measured in a laboratory setting (controlled temperature and moisture) in April and August 2022. C) Potential denitrification ratio (N_2O/N_2O+N_2) in percentage measured in a laboratory setting (n=5) under artificial atmosphere. *P*-values associated with the effect of eCO_2 and the effect of sampling date are indicated by P_{eCO2} and P_{time} respectively. Data are means + SE.

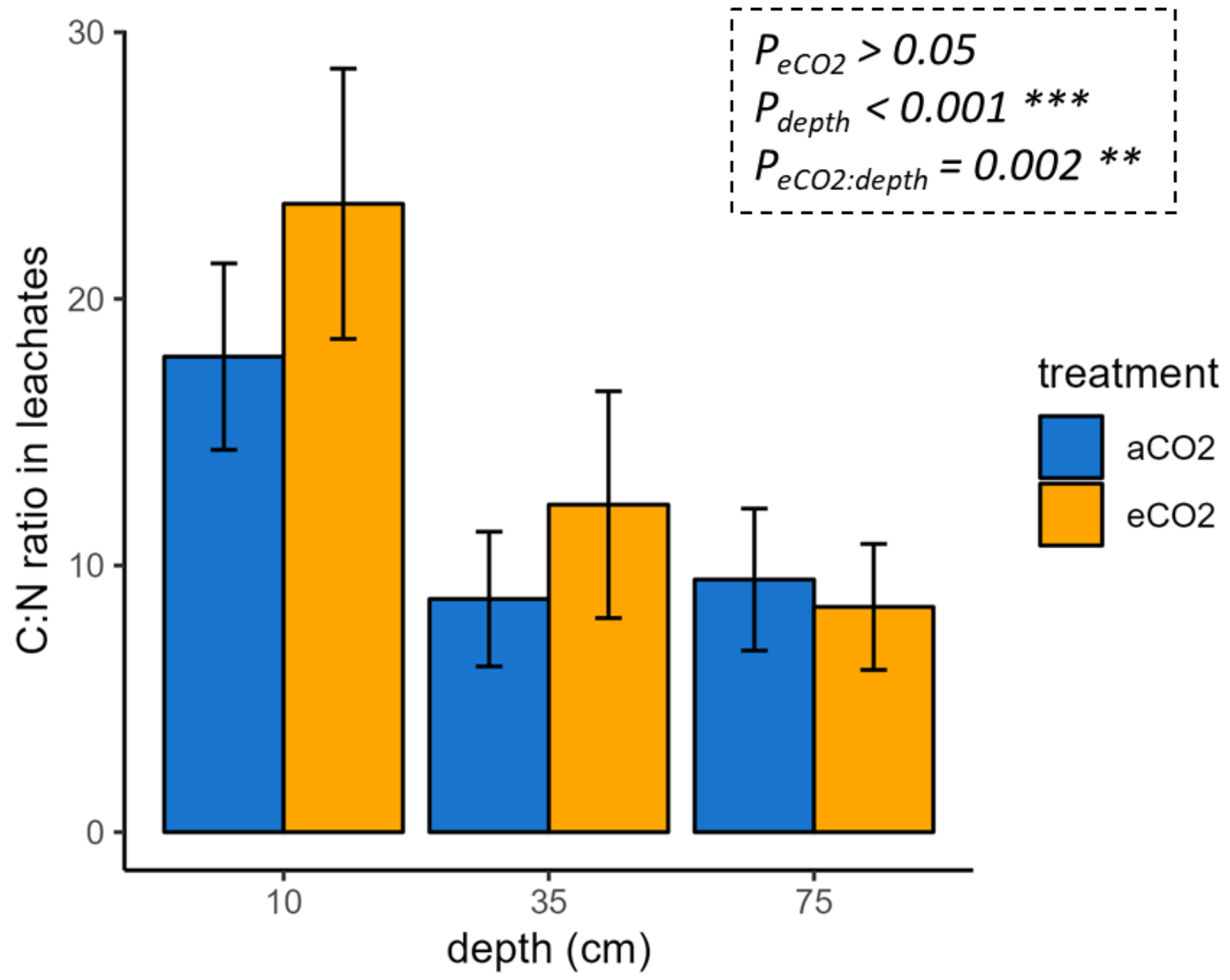

Fig. S7: Average of the C:N ratio measured in soil leachates at three different depths (10 cm, 35 cm and 75 cm) in 2023 using lysimeters. The *P*-values for the effect of eCO_2, depth and the interaction are detailed in the figure.

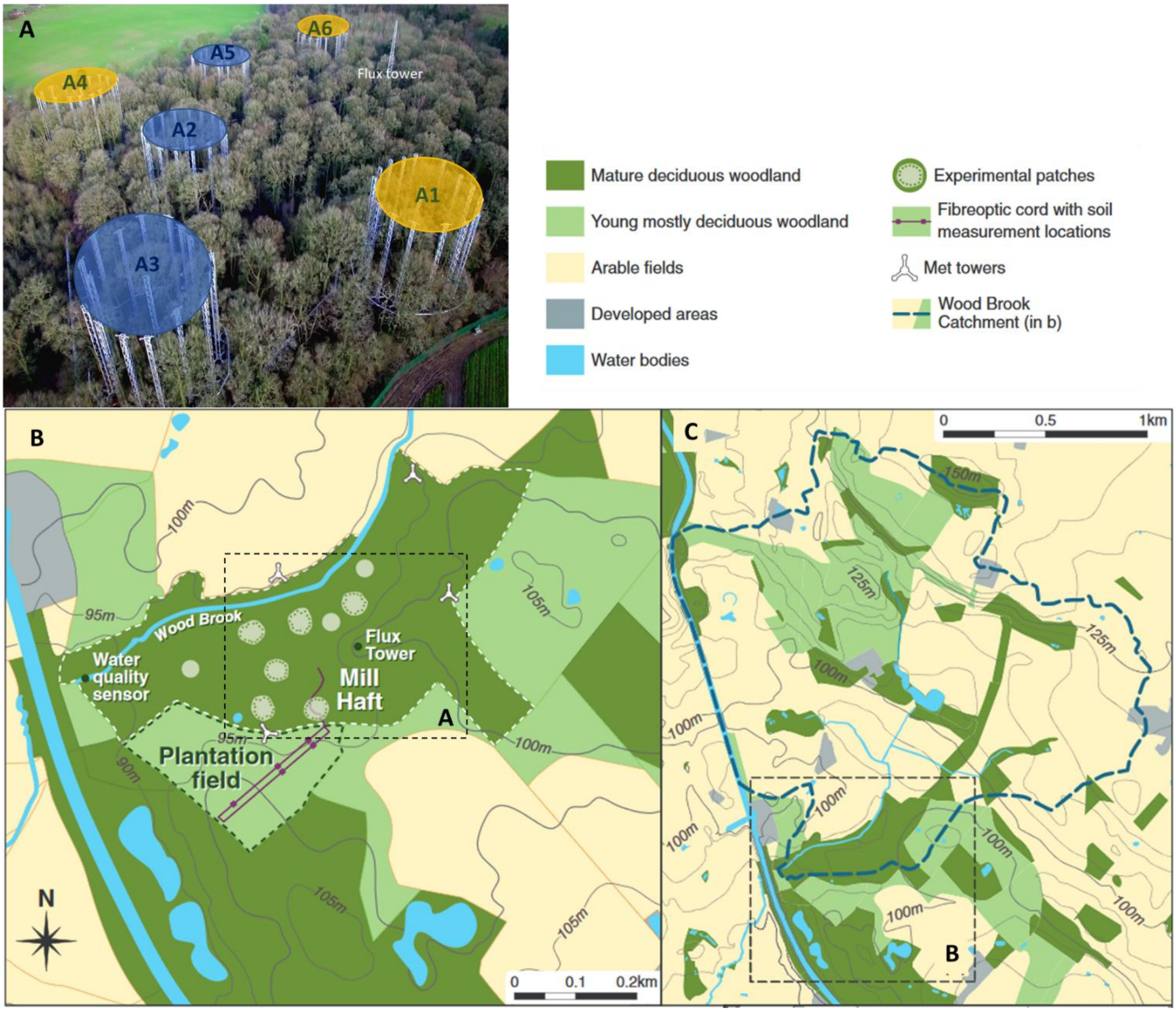

Fig. S8: A) Schematic of the BIFoR-FACE (fumigated arrays are represented in orange and ambient arrays are in blue) reproduced from Rumeau et al. (2024) (32). B) and C) Maps of BIFoR-FACE surrounding from MacKenzie et al. 2021 (41). Both panels are reproduced under a CC-BY 4.0 license (https://creativecommons.org/licenses/by/4.0/).
